# Motor impairments in Chinese toddlers with autism spectrum disorder and its relationship with social communicative skills

**DOI:** 10.3389/fpsyt.2022.938047

**Published:** 2022-10-14

**Authors:** Bingrui Zhou, Qiong Xu, Huiping Li, Ying Zhang, Dongyun Li, Ping Dong, Yi Wang, Ping Lu, Ye Zhu, Xiu Xu

**Affiliations:** Department of Child Healthcare, Children's Hospital of Fudan University, Shanghai, China

**Keywords:** autism spectrum disorder, motor impairment, communication, social interaction, autism severity

## Abstract

**Objective:**

Motor impairments are prevalent in children with autism spectrum disorder (ASD) and persistent across age. Our current study was designed to investigate motor deficits in Chinese toddlers with ASD and to explore the relationships between motor deficits and social communication skills.

**Methods:**

For this cross-sectional study, we recruited a total of 210 Chinese toddlers with ASD aged between 18 and 36 months in the study during December 2017 to December 2020. Griffiths Developmental Scales-Chinese (GDS-C), Autism Diagnostic Observation Schedule-Second Edition (ADOS-2) and Communication and Symbolic Behavior Scales Developmental Profile-Infant-Toddler Checklist (CSBS-DP-ITC) were administered in these toddlers to evaluate their development, social communicative skills, and autism severity. We compared the developmental and social communicational profiles of ASD toddlers in different gross and fine motor subgroups, and explored potential associated factors. The univariate generalized linear model tested the relationship of fine and gross motor skills and social communicative skills.

**Results:**

The prevalence of gross and fine motor deficits were 59.5 and 82.5%, respectively, which are almost equivalent in boys and girls. The motor impairments tended to be more severe with age in toddlers. After adjusting for age, sex, non-verbal development quotient (DQ) and restricted, repetitive behaviors, severer gross motor impairments were significantly related to higher comparison score of ADOS-2 and higher social composite score of CSBS-DP-ITC, without interactions with other variables. Meanwhile, lower fine motor skills were associated with more deficits of social communication and higher severity of ASD, also depending on non-verbal DQ. In the lower non-verbal DQ subgroup, both fine motor deficits and restricted repetitive behaviors (RRBs) might have effects on autism symptomology.

**Conclusion:**

Motor impairments are common in Chinese toddlers with ASD. Toddlers with weaker gross and fine motor skills have greater deficits in social communicative skills. Gross motor impairment might be an independent predictor of the severity of autism and social communication skills, while the effect of fine motor deficits might be affected by non-verbal DQ and RRBs of toddlers with ASD. We provide further justification for the inclusion of motor impairments in the early intervention for toddlers with ASD.

## Introduction

Autism spectrum disorder (ASD) is a lifelong developmental condition that is diagnosed during early childhood. ASD is characterized by persistent deficits in social communication and social interaction as well as restricted, repetitive patterns of behaviors, interests and activities. Motor impairment is present in 50–87% of children with ASD (1–4), and is a source of increasing concern. Delays in fundamental motor behaviors (e.g., motor milestones, walking patterns, reaching and aiming patterns, postural control, and complex motor planning) as well as socially- and cognitively-challenging motor behaviors (e.g., difficulties with interpersonal synchrony and cooperative actions, praxis and imitation difficulties) are seen in individuals with ASD (5–9) starting at a young age. Motor impairment increases with age and persists until age 15 (4).

Researchers have noted that children with ASD and weaker motor skills have significantly greater social communication and language skill deficits and higher calibrated autism severity scores, measured by both parent reporting and clinician administering (10–17). However, the evidence for specific associations between motor behavior and ASD symptomatology is less conclusive (18). One barrier to delineating such associations has been a lack of consensus regarding the nature of the interrelationships between motor skills, autism symptoms, and broader neurodevelopmental delay (19). Some studies suggest that motor impairments reflect a more severe level of neurodevelopmental vulnerability; the relationship between motor deficits and ASD symptoms is primarily mediated by differences in cognitive ability (5). A widely known study by Green et al. suggested that motor skills of autistic children with an IQ < 70 were more impaired than those with IQ more than 70 (1). Ramos et al. found significant associations between comorbid intellectual disability and motor impairment. Variance in performance IQ could explain 20.8% of the variance in motor skills (20). A large-scale study lent some support for this position; motor difficulties related to core ASD symptoms as well as cognitive ability and functional impairment (21). Additionally, motor skills, especially fine motor skills, were also positively associated with receptive and expressive language, which might mediate the relationship between motor skills and social interaction skills (17).

Still, there is evidence suggesting that the effects of motor deficits on social communication skills are above and beyond what can be attributed to cognitive ability alone (19). Another two recent large-scale studies of the SPARK dataset support a broad relationship between the motor domain and ASD-associated domains, regardless of intellectual ability. Ketcheson et al. found the effects of motor difficulties were independent of intellectual disability, and exhibited a medium effect size for social communication skills and a small effect size for restricted repetitive behaviors (22). Bhat also suggested the risk of motor deficits increases as social communication impairment and RRBs increase in severity (3). Recently, two meta-analyses reviewed 114 studies representing 6,423 autistic and 2,941 neurotypical individuals and revealed that individuals with ASD exhibit a large deficit in gross motor skills compared to neurotypical controls, regardless of age, sex, or cognitive ability, and the gross motor impairment is modestly associated with social impairment in individuals with ASD (14).

Some compelling studies on typical development suggests that motor skills are foundational to human learning, thus having a key role in shaping social communication behaviors from very early infancy (23, 24). An increasing number of studies have suggested that high-risk infants that are later diagnosed with ASD show signs of sensorimotor differences at very early age, even during their first year of life (25). Motor difficulties were predictive of later language skill deficiencies and/or an emerging autism phenotype (26–29). Related symptoms include weaker spontaneous leg movement, poor reaching-grasping movement (30), poor toy manipulation (31), and less frequent hand and knees crawling (32), some of which are fundamental motor milestones. These findings improved the recognition that motor skill is intrinsically linked to social communication and adaptive functioning (33). It has also been suggested that symptoms related to motor delays be added to the diagnostic criteria for ASD. Still, it has also been theorized that disruptions in early motor development, which are more easily identified by parents than deficiencies in other domains during infancy, are not specific to ASD, but rather reflect a broader neurodevelopmental risk status (29, 34).

In summary, most of the previous studies support that motor skills are related to social communication skills and symptomology of ASD. However, there is still a lack of consensus about the nature of the interrelationships between motor skills, autism symptoms, and broader neurodevelopmental disruptions (19). Meanwhile, most of the studies exploring the relationships between motor impairments and the core symptoms of ASD have been focused on preschoolers and school-aged children, or conducted in a large sample with a wide age range, while those in toddlers were relatively few (6, 12). Previous inconsistent findings are also fueled in part by variation in the age range, diagnostic groups, sources, and comorbidities of the children included in these works (15).

Therefore, in this study we further explore the relationship between motor impairments and autism symptomology in Chinese toddlers with ASD. We hypothesized that impaired motor skills are associated with autism severity, independent of verbal/non-verbal skills and repetitive behaviors. We also examined the interactive effects of motor skills and verbal/non-verbal DQ/RRBs to evaluate if the effects of motor skills on autism severity/social communication skills are dependent on language/performance (spatial ability skill).

## Methods

### Participants and procedures

In the cross-sectional study, participants were recruited in the outpatients of Department of Child Health Care and Developmental-Behavioral Pediatrics at the Children's Hospital of Fudan University between December 2017 and December 2020. Inclusion criteria were: (1) aged 18–36 months and full-term gestation (37–42 weeks); (2) diagnosed with ASD for the first time in our site without receiving interventions before; (3) confirmed diagnosis of ASD with the Autism Diagnostic Observation Schedule-Second Edition (ADOS-2). Exclusion criteria were the presence of chromosome abnormalities, congenital anomalies, or major neurological sensory impairments or disorders (e.g., cerebral palsy, microcephalus, macrocephalus, severe brain damage, uncorrected vision, or hearing loss).

Toddlers received clinical diagnoses of ASD by experienced developmental and behavioral pediatricians (Xiu Xu and Qiong Xu) according to the Diagnostic and Statistical Manual of Mental Disorders, 5^th^ edition (DSM-5), with a behavioral observation for no less than 30 min in the outpatient. Then, we made a reservation for candidate families and completed the collection of clinical data and assessments at the second consultancy. All assessments were completed in the same clinic setting with standardized assessment tools when toddlers were in good mental and emotional states. We first conducted Autism Diagnostic Observation Schedule-Second Edition (ADOS-2) assessment followed by Griffiths Developmental Scales-Chinese version (GDS-C) with a break of about 30 min for parents or caregivers to complete the Communication and Symbolic Behavior Scales Developmental Profile-Infant-Toddler Checklist (CSBS-DP-ITC). The two assessments for each toddler were performed by the same examiner. If a toddler did not complete both assessments, he/she would come to the clinic again in 1 week.

Toddlers received the assessments with a familiar parent/caregiver in the room. They were encouraged to remain seated at a child-sized testing table or on the parent's lap during the assessments, except for the gross motor tasks or some activities in ADOS-2. Parents/caregivers could not help their toddlers during most activities, except for several activities in ADOS-2 that can be partially completed by parents/caregivers.

This study was registered in the ClinicalTrials.gov database (https://clinicaltrials.gov/): Registry number NCT03847402. It was approved by the Institutional Review Board of the Children's Hospital of Fudan University, Shanghai, China. Written informed consent was obtained from the child's parents after they received a complete description of the study. The study was conducted in accordance with the ethical standards outlined in the Helsinki Declaration.

### Demographic information

Information including gestational week, birth weight, paternal and maternal education and childbearing ages were collected before the following assessments were administered.

### Griffiths developmental scales-Chinese version

The GDS-C (35, 36) is a standardized developmental assessment tool for children from birth to 8 years. There are six domains in the GDS-C. Domains A–E (locomotor, personal–social, language, eye–hand coordination, and performance) are administered to toddlers under 2 years old, while one more domain (F: practical reasoning) administered to children aged more than 2 years old. The GDS-C is derived and validated from the Griffith Mental Development Scales Extended Revised (37), and used in Chinese children with ASD (38). Developmental ages (DAs) originate from previously published norms and developmental quotients (DQs) are calculated with the formula DA/CA (chronological age) ^*^100. Li et al. (36) showed that the GDS-C is a reliable and valid neurodevelopmental assessment tool for Chinese children with ASD.

### Autism diagnostic observation schedule-second edition

ADOS-2 (39–41) is used for assessing ASD symptoms in a series of standardized, semi-structured activities across toddlers to adults with different verbal skills. The score of each item in the algorithm ranges from 0 to 2, with higher score indicating more severe deficits. The sum of social affect (SA) and restricted and repetitive behaviors (RRBs) is the total raw score, which can be transformed into a comparison score to compare directly across different modules (except Toddler Module) and over time. Higher comparison score indicates more severe core symptoms of ASD.

### Communication and symbolic behavior scales developmental profile-infant-toddler checklist (42)

The CSBS-DP-ITC is a parent-reported questionnaire widely used to screen for communication disorders including ASD in infants and toddlers aged 6–24 months. It consists of 24 items divided into three subscales (social, speech, and symbolic composite) with an open question at the end. Score of each item ranges from 0 to 2/3/4. Lower total score indicates poorer social communication skills. Changes in the raw scores of each subscale and the total score partly reflect changes in the child's social communication skills. The scale is used to independently describe the social communication skills *via* parents' report in their daily lives.

### Quality control

Three authors of the study received the advanced training of the ADOS-2. One author acquired the qualification for ADOS. The inter-rater reliabilities of scoring between each of other assessors and the qualified assessor were over 0.8. All authors received the GDS-C training and acquired the qualification.

### Data analysis

Participants were categorized into three subgroups according to their gross and fine motor DQs: normal (DQ > 85), moderate (DQ = 70–85), and low (DQ < 70). The demographic information and scores of GDS-C, ADOS-2, and CSBS-DP-ITC were compared between different groups or subgroups using chi-squared, *t*-test, and analysis of variance (or a non-parametric test), with an a priori alpha level of 0.05. To account for multiple comparisons, a Bonferroni-adjusted α = 0.05/3 = 0.017 was used for *post-hoc* analyses between each two subgroups of gross and fine motor skills after analysis of variance, while all pairwise was used for *post-hoc* analyses after non-parametric test (Kruskal-Wallis 1-way ANOVA).

Linear regression analysis was performed to identify the independent determinants of higher comparison score/SA score of ADOS-2 and scores of CSBS-DP-ITC. Candidate variables with a *p*-value < 0.15 in linear regression were included in univariate GLM. Also, we compared the differences in autism severity and social communication skills between boys and girls to decide whether sex should be included or not as a potential variable. Then, we tested relationships between gross and fine motor skills as measured with the GDS-C with autism symptomology as measured by the social affect, and comparison score of the ADOS-2 as well as the scores of CSBS-DP-ITC. We used linear regression or univariate generalized linear model (GLM) depending on the distribution of the dependent variables. Data analysis was performed using Statistical Package for the Social Sciences (SPSS) version 22.0 (IBM, Armonk, NY, USA).

## Results

### Descriptive results

A total of 210 Chinese toddlers with ASD (183 boys and 27 girls) with a mean age of 24.40 months old and a standard deviation of 3.26 months participated in the study based on the inclusion and exclusion criteria. The demographic characteristics of the participants are shown in Table 1.

**Table 1:** Descriptive characteristics.

**Variable**	**Mean ±SD/Frequency**
Age (months)	24.40 ± 3.26
Sex	183 boys, 27 girls
Paternal childbearing age	31.55 ± 4.77
Maternal childbearing age	29.75 ± 4.04
Paternal education	30 graduate/professional, 84 college, 23 high school, 19 primary/middle school, 54 unspecified
Maternal education	21 graduate/professional, 91 college, 24 high school, 20 primary/middle school, 54 unspecified
Gestational weeks	39.29 ± 1.19
Birth Weight (g)	3415.14 ± 418.60
**GDS-C (*****n*** **=** **210)**
A: Gross motor	82.53 ± 12.29
B: Personal-social	57.29 ± 16.54
C: Language	41.00 ± 17.81
D: Hand-eye coordination	69.87 ± 16.80
E: Performance	71.13 ± 21.14
**ADOS-2 (*****n*** **=** **139)**
SA	16.55 ± 3.15
RRBs	2.02 ± 1.38
Total score	18.58 ± 3.75
CS (*n* = 65)	6.57 ± 1.45
**CSBS-DP-ITC**
Social	17.43 ± 8.84
Speech	5.72 ± 2.79
Symbolic play	8.71 ± 3.15

GDS-C, Griffiths Developmental Scale-Chinese; A, Gross Motor domain of the GDS-C; B, Personal-Social; C, Language; D, Hand-Eye Coordination; E, Performance; ADOS-2, Autism Diagnostic Observation Schedule, 2nd edition; SA, Social Affect of the ADOS-2; RRBs, Restricted and Repetitive Behavior of the ADOS-2; Total, total score of the ADOS-2; CS, ADOS-2 comparison score; CSBS-DP-ITC, Communication and Symbolic Behavior Scales Developmental Profile-Infant-Toddler Checklist.

We investigated the distributions of normal (DQ > 85), moderate (DQ = 70–85), and low (DQ < 70) levels of gross and fine motor skills, and compared the developmental profile, autism symptomatology, and social communication skills reported by parents (Table 2). Overall, both the rate and severity of fine motor deficits were higher than that of gross motor deficits in these toddlers with ASD. There were no significant differences in the distribution of gross and fine motor deficits in boys and girls [for gross motor: $\chi_{\left(2,\text{N}=210\right)}^{2}$ = 0.481, *p* = 0.785; for fine motor: $\chi_{\left(2,\text{N}=210\right)}^{2}$ = 2.493, *p* = 0.287].

**Table 2:** Developmental scores and ASD symptoms of participants with different degrees of motor dysfunction.

	**Gross motor**			**F/χ^2^/H**	***p-*value**	**Fine motor**			**F/χ^2^/H**	***p*-value**
	**Normal**	**Mod**	**Low**			**Normal**	**Mod**	**Low**		
***n* (%)**	**85 (40.5%)**	**95 (45.2%)**	**30 (14.3%)**			**40 (19.1%)**	**63 (30.0%)**	**107 (50.9%)**		
**Sex**
Boys (%)	75 (41.0%)	83 (45.4%)	25 (13.7%)	0.483	0.785^c^	32 (17.5%)	57 (31.1%)	94 (51.4%)	2.493	0.287^c^
Girls (%)	10 (37.0%)	12 (44.4%)	5 (18.5%)			8 (29.6%)	6 (22.2%)	13 (48.1%)		
Age	22.95 ± 3.35	25.08 ± 2.59	26.42 ± 3.06	19.293	< 0.001^*^^,^ ^a^	23.30 ± 4.00	24.30 ± 2.63	24.88 ± 3.17	3.625	0.029^*^^,^ ^a^
**GDS-C**
A	94.32 ± 7.82	77.84 ± 3.85	63.98 ± 5.29	175.262	< 0.001^*^^,^ ^b^	91.67 ± 11.73	84.26 ± 10.85	78.10 ± 11.20	22.475	< 0.001^*^^,^ ^b^
B	63.21 ± 17.39	56.13 ± 14.30	44.13 ± 12.05	32.643	< 0.001^*^^,^ ^b^	75.08 ± 16.79	57.93 ± 13.23	50.25 ± 12.78	47.537	< 0.001^*^^,^ ^b^
C	45.05 ± 18.23	39.99 ± 18.24	32.72 ± 11.09	20.627	< 0.001^*^^,^ ^b^	59.22 ± 22.13	40.08 ± 17.04	34.73 ± 10.47	37.253	< 0.001^*^^,^ ^b^
D	76.11 ± 16.19	68.54 ± 15.16	56.39 ± 14.94	18.471	< 0.001^*^^,^ ^a^	93.81 ± 6.26	77.78 ± 4.02	56.26 ± 9.60	392.772	< 0.001^*^^,^ ^a^
E	78.14 ± 21.63	68.21 ± 18.80	60.53 ± 20.77	10.182	< 0.001^*^^,^ ^a^	94.15 ± 13.74	75.55 ± 15.85	59.93 ± 18.08	64.438	< 0.001^*^^,^ ^a^
**ADOS-2**
*n* (%)	65 (46.8%)	57 (41.0%)	17 (12.2%)			26 (18.7%)	39 (28.1%)	74 (53.2%)		
SA	16.06 ± 3.23	16.58 ± 3.39	16.66 ± 2.87	0.707	0.704^b^	15.23 ± 3.25	16.18 ± 3.36	17.22 ± 2.85	10.181	0.006^*^^,^ ^b^
RRBs	2.12 ± 1.62	1.98 ± 1.42	2.04 ± 1.27	0.239	0.892^b^	1.96 ± 1.39	1.74 ± 1.29	2.19 ± 1.40	2.696	0.260^b^
Total	18.17 ± 3.75	18.57 ± 4.10	18.70 ± 3.38	0.312	0.857^b^	17.19 ± 3.97	17.92 ± 3.91	19.41 ± 3.41	8.892	0.012^*^^,^ ^b^
CS	6.67 ± 1.43	6.87 ± 1.66	6.30 ± 1.26	1.904	0.386^b^	6.00 ± 1.82	6.56 ± 1.41	6.72 ± 1.36	3.022	0.221^b^
**CSBS-DP-ITC**
Social	17.78 ± 8.52	17.56 ± 9.32	15.52 ± 8.08	0.971	0.617^b^	18.91 ± 9.45	19.49 ± 8.33	15.28 ± 8.54	11.307	0.004^*^^,^ ^b^
Speech	5.79 ± 3.09	5.90 ± 2.69	4.68 ± 1.70	2.927	0.232^b^	6.97 ± 3.68	5.68 ± 2.56	5.19 ± 2.30	6.014	0.05^*^^,^ ^b^
Symbolic	9.29 ± 3.30	8.63 ± 2.97	6.89 ± 2.77	8.586	0.014^*^^,^ ^b^	10.48 ± 3.28	9.28 ± 2.99	7.50 ± 2.72	22.237	< 0.001^*^^,^ ^b^

GDS-C, Griffiths Developmental Scale-Chinese; A, Gross Motor domain of the GDS-C; B, Personal-Social; C, Language; D, Hand-Eye Coordination; E, Performance; ADOS-2, Autism Diagnostic Observation Schedule, 2nd Edition; SA, Social Affect; RRBs, Restricted and Repetitive behaviors; Total, total score of the ADOS-2; CS, ADOS-2 comparison score; CSBS-DP-ITC, Communication and Symbolic Behavior Scales Developmental Profile-Infant-Toddler Checklist; Social, the social composite score of CSBS-DP-ITC; Speech, the speech composite score of CSBS-DP-ITC; Symbolic, the symbolic play composite score of CSBS-DP-ITC.

a One-factor ANOVA test.

b Nonparametric test.

c Chi-square test.

* *p* < 0.05.

#### Developmental profiles and autism symptomology of different motor subgroups

We further examined the demographic characteristics and raw scores of the standardized assessments within the three motor subgroups (Table 2). Toddlers with normal motor skills were significantly younger than those with low motor skills, both in gross and fine motor domain (gross motor: F_(2, 207)_ = 19.293, *p* < 0.001; fine motor: F_(2, 207)_ = 3.625, *p* = 0.029).

With regard to the developmental profile shown in the Table 2, the three gross and fine motor subgroups demonstrated significantly different levels of personal-social, language, and performance skills (all *p*-values < 0.001, with a Bonferroni correction), respectively. *Post-hoc* analyses revealed significant differences between each two subgroups (normal vs. moderate, moderate vs. low, and normal vs. low, in gross and fine motor skills, respectively).

With respect to ASD symptoms, social affect (*H* = 10.181, *p* = 0.006) and overall scores (*H* = 8.892, *p* = 0.012) were markedly different among the three fine motor subgroups. The social affect scores of toddlers with normal fine motor skill were significantly lower than those with low fine motor skills (all-pairwise adjusted *p* = 0.002). However, the scores of these two subgroups were equivalent to those of the moderate-low subgroup.

The age and developmental performance of the gross motor subgroups were similar to those of the fine motor subgroups. However, the ADOS-2 scores among the gross motor subgroups showed no significant difference (all *p*-values > 0.05) (Table 2).

#### Parent-reported communication and symbolic behaviors of different motor subgroups

Three fine motor subgroups showed significantly different scores of all of three subscales of CSBS-DP-ITC (Table 2). Further analyses identified the low subgroup got significantly lower scores than normal and moderate-low subgroups in social composite, while normal subgroup performed better than the other two subgroups in speech. Moreover, scores of symbolic play between each two among the three fine motor subgroups were significantly different (normal > moderate > low). However, the differences among gross motor subgroups were not so marked as those among fine motor subgroups.

### Relationship between gross motor skills and ASD symptoms

The results of linear regression analyses were shown in Table 3. Candidate variables with a *p*-value < 0.15 were included in the univariate GLM.

**Table 3:** Linear regression analyses of comparison score and SA score of ADOS-2, and social composite score of CSBS-DP-ITC.

**Dependent variable**	**Standardized beta**	***t***	***p*-value**
**CS**			
Age	0.097	0.773	0.442
Gross motor	−0.139	−1.402	**0.146**
Verbal DQ	−0.181	−1.461	**0.140**
Fine motor	−0.214	−1.739	**0.087**
Non-verbal DQ	−0.319	−2.674	**0.010**
RRBs	0.515	4.767	**< 0.001**
**SA**			
Age	0.046	0.538	0.592
Gross motor	−0.110	−0.116	0.908
Verbal DQ	−0.383	−4.850	**< 0.001**
Fine motor	−0.268	−3.259	**0.001**
Non-verbal DQ	−0.262	−3.179	**0.002**
RRBs	0.261	3.170	**0.002**
**Social**			
Age	0.046	0.595	0.552
Gross motor	0.097	1.511	**0.136**
Verbal DQ	0.157	2.070	**0.040**
Fine motor	0.201	2.668	**0.008**
Non-verbal DQ	0.170	2.242	**0.026**
RRBs	0.114	1.712	**0.141**

GDS-C, Griffiths Developmental Scale-Chinese; ADOS-2, Autism Diagnostic Observation Schedule, 2nd Edition; CS, ADOS-2 comparison score; SA, Social Affect score of ADOS-2; RRBs, Restricted and Repetitive behaviors score of ADOS-2; Communication and Symbolic Behavior Scales Developmental Profile-Infant-Toddler Checklist; Social, the social composite score of CSBS-DP-ITC; Gross motor, DQ of gross motor domain in GDS-C; Verbal DQ, DQ of language domain in GDS-C; Fine motor, DQ of hand-eye coordination domain in GDS-C; Non-verbal DQ, DQ of performance domain in GDS-C. Bold values means the variables with *p*-values < 0.15 in linear regression analyses will be included in the generalized linear model.

We tested the relationship between categorical gross motor skill and the comparison score and social affect score of the ADOS-2 using a univariate GLM. Based on the linear regression analyses, verbal DQ, performance (non-verbal DQ), and RRBs were included in the model as covariates. Because of the significant difference in comparison score of ADOS-2 between boys and girls (*t*-test, *t* = 2.335, *p* = 0.044), sex was also included as a potential factor.

Our results identified significant relationships between gross motor impairment and comparison score of ADOS-2 (Table 4). Using regression coefficients (B), toddlers without gross motor impairment, regardless of other differences, had significantly milder autism symptoms (the low subgroup as a reference, for the normal subgroup: B = −6.290, Wald's χ2 = 9.264, *p* = 0.002; for the moderate subgroup: B = −4.413, Wald's χ2 = 4.633, *p* = 0.031). Furthermore, the comparison score was also significantly associated with sex (B = 1.250 for girls as a reference, Wald's χ2 = 6.178, *p* = 0.013), performance (B = −0.036, Wald's χ2 = 5.796, *p* = 0.016) and RRBs (B = 0.264, Wald's χ2 = 7.044, *p* = 0.008). Verbal DQ showed no effect on the comparison score (Wald's χ2 = 0.226, *p* = 0.634). We further examined the interactive effects between gross motor and verbal DQ/performance/RRBs. The interactions between gross motor and verbal DQ/performance/RRBs were not statistically significant for the ADOS-2 comparison score (Wald's χ2 = 3.336, *p* = 0.189 for verbal DQ, Wald's χ2 = 4.880, *p* = 0.087 for performance, Wald's χ2 = 4.167, *p* = 0.114 for RRBs).

**Table 4:** Relationships between gross motor skills and social communication skills using generalized linear model.

**Parameter**	**B**	**SE**	**Wald χ^2^**	***p*-value**
**Comparison score**				
Sex-boys	1.250	0.503	6.178	0.013^a^
Sex-girls (reference)	–	–	–	–
Gross motor subgroup-normal	−6.290	2.066	9.264	0.002^b^
Gross motor subgroup-moderate	−4.413	2.050	4.633	0.031^b^
Gross motor subgroup-low (reference)	–	–	–	–
Verbal DQ	−0.047	0.035	2.073	0.150^a^
Non-verbal DQ	−0.036	0.015	5.796	0.016^a^
RRBs	0.264	0.017	7.044	0.008^a^
**Social affect**				
Sex-boys	−1.100	0.745	2.184	0.139^a^
Sex-girls (reference)	–	–	–	**–**
Gross motor subgroup-normal	−1.813	3.923	0.214	0.644^b^
Gross motor subgroup-moderate	−1.356	3.916	0.120	0.729^b^
Gross motor subgroup-low (reference)	–	–	–	**–**
Verbal DQ	−0.085	0.072	1.390	0.238^a^
Non-verbal DQ	0.001	0.035	0.002	0.968^a^
RRBs	0.096	0.467	0.043	0.836^a^

a α = 0.05.

b A Bonferroni-adjusted α = 0.05/3 = 0.017. *p < 0.05 or 0.017.

GDS-C, Griffiths Developmental Scale-Chinese; ADOS-2, Autism Diagnostic Observation Schedule, 2nd Edition; Comparison Score, ADOS-2 comparison score; Social Affect, Social Affect score of ADOS-2; RRBs, Restricted and Repetitive behaviors score of ADOS-2; Gross motor, DQ of gross motor domain in GDS-C; Verbal DQ, DQ of language domain in GDS-C; Fine motor, DQ of hand-eye coordination domain in GDS-C; Non-verbal DQ, DQ of performance domain in GDS-C.

We found no significant relationships between gross motor skills and the social affect score of ADOS-2.

### Relationship between fine motor skills and ASD symptoms

Similarly, we chose the social affect and comparison scores of the ADOS-2 as dependent variables, categorical fine motor skills and sex as fixed factors, and language (verbal DQ), performance (non-verbal DQ), and RRBs as covariates in a univariate GLM. After adjusting for the effects of sex (B = 1.162 for girls as a reference, Wald's χ2 = 3.825, *p* = 0.042), performance (B = −0.027, χ^2^ = 3.921, *p* = 0.048), and RRBs (B = 0.337, χ^2^ = 7.217, *p* = 0.007), fine motor impairment severity was significantly associated with ADOS-2 comparison score (the low subgroup as a reference, for normal subgroup: B = −4.474, Wald's χ^2^ = 7.732, *p* = 0.005; for moderate subgroup: B = −1.874, Wald's χ^2^ = 4.627, *p* = 0.031, see Table 5). Autism symptoms tended to be milder in toddlers with normal fine motor skills compared with those who had low fine motor development (B < 0, with the low group as a reference). The difference between normal and moderate subgroups was not significant (*p* = 0.313).

**Table 5:** Relationships between fine motor skills and social communication skills using generalized linear model.

**Parameter**	**B**	**SE**	**Wald χ^2^**	***p*-value**
**Comparison score**				
Sex-boys	1.162	0.510	3.825	0.042^*^^,^ ^a^
Sex-girls (reference)	–	–	–	–
Fine motor subgroup-normal	−4.474	1.128	7.732	0.005^*^^,^ ^b^
Fine motor subgroup-moderate	−1.874	0.776	4.627	0.031^b^
Fine motor subgroup-low (reference)	–	–	–	**–**
Verbal DQ	0.010	0.017	0.339	0.561^a^
Non-verbal DQ	−0.027	0.013	3.921	0.048^*^^,^ ^a^
RRBs	0.337	0.126	7.217	0.007^*^^,^ ^a^
**Social affect**				
Sex-boys	−1.156	0.787	2.157	0.142^a^
Sex-girls (reference)	–	–	–	**–**
Fine motor subgroup-normal	−4.378	3.718	0.861	0.353^b^
Fine motor subgroup-moderate	−2.972	2.945	1.018	0.313^b^
Fine motor subgroup-low (reference)	–	–	–	**–**
Verbal DQ	−0.031	0.035	0.779	0.377^a^
Non-verbal DQ	−0.002	0.019	0.014	0.905^a^
RRBs	0.322	0.232	1.929	0.165^a^

a α = 0.05.

b A Bonferroni-adjusted α = 0.05/3 = 0.017.

* *p* < 0.05 or 0.017.

GDS-C, Griffiths Developmental Scale-Chinese; ADOS-2, Autism Diagnostic Observation Schedule, 2nd Edition; Comparison Score, ADOS-2 comparison score; Social Affect, Social Affect score of ADOS-2; RRBs, Restricted and Repetitive behaviors score of ADOS-2; Gross motor, DQ of gross motor domain in GDS-C; Verbal DQ, DQ of language domain in GDS-C; Fine motor, DQ of hand-eye coordination domain in GDS-C; Non-verbal DQ, DQ of performance domain in GDS-C.

**Figure 1:**
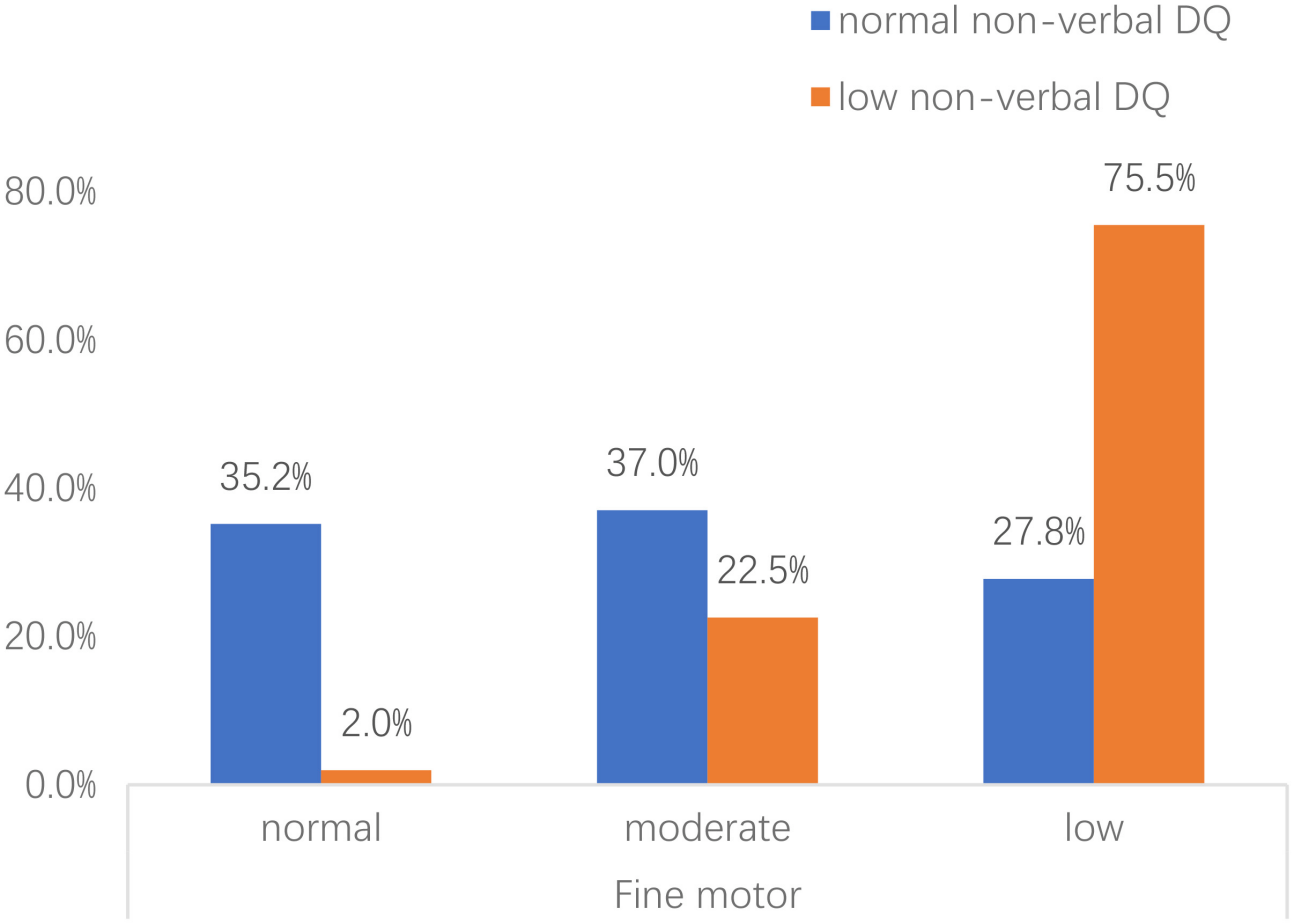
Proportion of toddlers with ASD with risk for fine motor impairment across different non-verbal DQ subgroups. Non-verbal DQ: measured by performance domain of Griffiths Development Scales-Chinese (GDS-C). Fine motor: normal, the DQ of hand-eye coordination domain>85; moderate, the DQ of hand-eye coordination domain ranges 70–85; low, the DQ of hand-eye coordination domain < 70.

Importantly, we found an interactive effect between fine motor skills and performance (non-verbal DQ) (Wald's χ^2^ = 5.601, *p* = 0.018). The severity of general fine motor impairment was significantly higher in toddlers with low non-verbal DQ than that of toddlers with normal non-verbal DQ [$\chi_{\left(2,\text{N}=210\right)}^{2}$ = 57.508, *p* < 0.001] (Figure 1). Further analyses were conducted in subgroups of impaired and unimpaired performance, and suggested that in the impaired subgroup of performance, the effect of fine motor impairment on the ADOS-2 comparison score was significantly related to RRBs (Wald's χ^2^ = 3.382, *p* = 0.033).

In the other GLM model, fine motor impairment severity had no effect on the social affect score of the ADOS-2.

### Relationship between motor skills and parent-reported social-communication skills

We used linear regression models to examine the effects of motor skills on parent-reported social-communicational skills using the CSBS-DP-ITC. Social composite score and total score of CSBS-DP-ITC were used as dependent variables. Age at enrollment, scores of five subscales of the GDS-C, and RRBs were included as independent variables. Given the correlations between the scores in each subscale of the GDS-C, we filtered the variables using a stepwise analysis (Table 6).

**Table 6:** Linear regression model of CSBS total score and social composite.

**Parameter**	**Adjusted *R*^2^**	**Standardized B**	***t***	***p-*value**
**CSBS total score**				
Age	0.541	0.183	2.024	0.046
Gross motor		0.267	2.757	0.007
Fine motor		0.245	2.547	0.012
Verbal DQ		0.284	3.067	0.003
**Social composite**				
Age	0.410	0.136	1.973	0.039
Gross motor		0.239	2.530	0.013
Fine motor		0.289	3.057	0.003

CSBS total score, the total score of Communication and Symbolic Behavior Scales-Developmental Profile-Infant-Toddler Checklist; Gross motor, DQ of gross motor domain in GDS-C; Verbal DQ, DQ of language domain in GDS-C; Fine motor, DQ of hand-eye coordination domain in GDS-C.

In the model with the social composite as the dependent variable, age at enrollment and the DQs of the gross and fine motor (hand-eye coordination) subscales of the GDS-C were included in the model (adjusted *R*^2^ = 0.410). In the model with the total score as the dependent variable, age at enrollment, the DQs of gross motor, fine motor, and language (verbal DQ) were included in the model (adjusted *R*^2^ = 0.541). The detail information of the models is shown in Table 6 (all *p*-values < 0.05). The above two models passed the collinearity diagnostics (VIF < 3).

## Discussion

The present study investigated the prevalence of motor impairment in Chinese toddlers with ASD and explored the relationship between autism symptoms and gross and fine motor skills. It considered the potential effects of sex, verbal and nonverbal ability, and repetitive behaviors in this analysis.

Previous studies of motor impairments in ASD mostly conducted in elder children (mainly in preschoolers and school-aged children over 3 years) or in a large sample with a wide age range (2–17 years old). What this study adds to the literature is an analysis of the gross and fine motor skills, respectively, in a cross-sectional sample of toddlers with ASD in a more concentrated age range in China. Our participants were diagnosed with ASD for the first time without receiving interventions before, to minimize other possible confounders.

In the current study, we found that greater motor impairments were associated with higher severity of ASD and also with more deficits in social communication skills. However, the effects of gross motor impairments were independent of non-verbal DQ and RRBs, while the effects of fine motor impairments were related to non-verbal DQ, but not RRBs. Then, we further explored the association in the subgroups of normal and low non-verbal DQ, respectively, and found a significant interactive effect between fine motor skills and RRBs to the calibrated severity score of ADOS-2 in the low non-verbal DQ group.

### Prevalence of motor impairment in toddlers with ASD

The prevalence of gross and fine motor impairment (DQ < 85) in toddlers with ASD aged between 18 and 36 months in the present work was 59.5 and 80.9%, respectively, which was similar to those found in a previous study by MacDonald et al. (12). However, MacDonald et al. also included some non-ASD toddlers (23/159) in their sample. The prevalence of motor impairments in elder children with ASD ranged from 50 to 86.9% (1, 4, 21, 43). Our findings supported high prevalence of motor difficulties in younger toddlers with ASD using Griffiths Developmental Scales-Chinese version to objectively assess their gross and fine motor skills, which might be more accurate than those assessed via parent reporting or a retrospective clinical records review (44).

Our results also suggest that the gross and fine motor skills of young toddlers with ASD become progressively more delayed with age, which was consistent with the findings of previous studies (6, 45). As a primary element of active play in young toddlers, movement skills will develop along with other skills including social skills, an understanding of the world, daily living skills, and adaptive behavior during playing games with peers (46, 47). During the play, toddlers explore and imitate motor skills independently or from peers. Both the play and social engagement are challenging for children with ASD. Therefore, Lloyd et al. proposed that there might be a malign cycle where poor motor skills constrain social interactions, and poor social interactions constrain motor skill development (6). Early intervention would improve social communication skills of toddlers with ASD, break the malign cycle, and simultaneously ameliorate their motor impairments.

We found no significant differences in gross or fine motor skills between boys and girls. These results are consistent with a recent study of Duvall et al. on a large sample of toddlers with ASD that directly evaluated motor skills using the motor subscales of Mullen Scales of Early Learning (48). However, prior studies based on both direct evaluation (49–51) and parental reports (52) found that girls had significantly stronger fine motor skills but worse gross motor skills than boys. Contrasting findings are also reported by Matheis et al., who used the Battelle Developmental Inventory, 2nd Edition (BDI-2) to find that in toddlers, girls with ASD had greater motor impairments than boys after controlling for cognitive ability (53). Brain imaging studies may provide new insights into this issue. An MRI study (54) performed on ASD children with a mean age between 9 and 10 years suggested that the gray matter in the motor system, including the regions involved in motor planning and execution, could significantly differentiate girls and boys with ASD with a high classification accuracy. It should be noted that the above-mentioned studies varied in the size and age of their patient groups. A larger sample with longitudinal development profile and brain imaging follow-up might be a promising way to further explore sex differences in ASD.

### Relationships between motor skills and autism severity

Our results partially support our hypothesis that motor impairments frequently exist in children with ASD and appear to be associated with autism severity (12) and social behaviors (55). We found that both gross and fine motor delays, especially severe delays (DQ < 70), could aggravate autism severity. Further, sex, non-verbal performance, and RRBs were predictive of greater overall ASD severity.

There were no interactions between each variable in the model of gross motor as the dependent variable, indicating that gross motor skills are independently associated with autism severity as measured with the ADOS-2. The social score of the CSBS-DP-ITC was also associated with the DQs of gross motor and hand-eye coordination (fine motor), regardless of age. These factors did not have a significant impact on the social effect score of the ADOS-2. We considered this to be reasonable because these scores are not standardized between different modules of the ADOS-2. Interestingly, we found that the effect of fine motor skills on autism severity was associated with non-verbal DQ (performance). Furthermore, in the impaired subgroup of non-verbal DQ, severity of RRBs would also interact with the effect of fine motor impairment on the severity of autism.

Our outcomes were partially similar to those of previous studies (5, 11, 12, 20–22, 49), most of which were conducted in preschoolers or school-age children. Some prior works found that children with ASD and cognitive impairment have significantly higher rates of and more profound motor impairment than those with ASD alone (21), possibly indicating that motor deficits reflect a more severe degree of neurodevelopmental vulnerability (20). However, regardless of their IQ scores, more gross and fine motor challenges are present across the entire spectrum of children with ASD than in typically developing children (5). Further, IQ can have minimal impact on motor skills (21). Ketcheson et al. recently reported that significantly greater core ASD deficits were identified in children who were either at-risk for a Development Coordination Disorder (DCD) and who had an intellectual disability (ID) (22). However, the effects of DCD were independent of ID. Core ASD symptoms were more strongly associated with motor difficulties than with cognitive ability. A small effect size was also found for restricted repetitive behaviors as measured with the RBS-R, which was consistent with our findings. Craig et al. found that fine motor skills were predictive of impaired social affect in boys but not in girls, deducing that motor skills might be the core feature of sex-related differences in ASD (49). However, one work has shown that while motor challenges were more prevalent in children with ASD compared with other groups, sensory symptom severity and IQ predicted motor performance better than the diagnosis group did (18). Our results further suggest that gross motor impairment may be independently predictive of autism severity, while fine motor deficit may contribute to higher deficits of social communication and higher severity of ASD which are partially mediated by visual spatial deficits and RRBs. Few studies have reported an association between motor skills and autism severity in young toddlers, and our study supported and expanded upon these results.

Some genetic and brain imaging studies tend to support that motor impairment might be a predictor of ASD. Serdarevic et al. calculated polygenic risk scores (PRSs) for ASD using genome-wide association study summary statistics, finding that higher PRSs for ASD were associated with suboptimal overall infant neuromotor development, especially low muscle tone (56). The genetic correlation between overall motor development and autistic traits was significant (0.35). Functional MRIs in school-age children with ASD (57) confirmed that visual-motor functional connectivity is disrupted in ASD, which might result in diminished integration of visual consequences with motor output. Temporal incongruity between visual and motor systems was predictive of the social deficit severity of children with ASD. Floris et al. reviewed the literature on atypical hemispheric specialization in the motor domain, and concluded that atypical structural and functional lateralization had the potential to serve as a neural marker of the atypical development of children with ASD, particularly with respect to the motor domain (58). In the future, studies using direct measurements of motor and social communication ability combined with genetic or imaging techniques will be promising to explore the nature of the interrelationships between motor skills, autism symptoms, and broader neurodevelopmental disorders.

### Limitations

There were several limitations to our study. First, the proportion of girls in our sample was relatively low, resulting in lower quality comparisons of the developmental profiles of boys and girls. Some studies have found evidence of differences in the development profile, social-communicational skills, and restricted repetitive behaviors of children with ASD between sexes. While sex was included in our ADOS-2 comparison score model, the raw data between boys and girls was equivalent. Second, we performed limited assessments in our sample. The Peabody Motor Development Scale would be appropriate for evaluating the gross and fine motor skills of toddlers. The Repetitive Behavior Scale-Revised (RBS-R) is also widely used for children with ASD. These evaluations would create a more comprehensive profile for each participant. Furthermore, not all researchers who performed the ADOS-2 had the qualification. We used module 1 or 2 of ADOS-2 instead of the toddler module in our sample because we had not received the training for the toddler module during the study. As an outcome measure, we explored the relationship between motor skills and social affect score, which is usually not compared directly between different modules. Third, due to the lack of appropriate scales for the toddlers in this study, we did not screen for developmental coordination disorder (DCD) nor exclude participants with potential DCD from those only with ASD. Besides, the CSBS-DP-ITC was completed by parents and could be prone to multiple forms of bias.

## Conclusion and future directions

Based on the results of the current study, we consider that generalized gross motor differences might be an independent factor of autism severity, above and beyond what can be attributed to visual spatial ability and RRBs, while the effects of fine motor impairments on autism severity might be partially affected by the deficit in visual spatial ability and RRBs, in Chinese toddlers with a concentrated age range. More elaborate research using a large, representative, and cross-conditioned sample may be able to further evaluate motor performance variability as a potential diagnostic and prognostic marker of ASD. More advanced techniques for collecting and analyzing motor data, such as gait analysis (59), 3D motion capture, wearable sensors (60), digital phenotyping through computer vision (61), and machine learning (62) now allow for detailed kinematic analysis and show promise for identifying novel motor biomarkers for the presence and severity of ASD. These approaches have particular significance for ASD because of their ability to capture subtle patterns of intra- and inter-individual variability and preserve the entirety of motor behavior rather than reducing it to a broad rating or summary score.

In general, the number of children with ASD who held a dual diagnosis related to motor impairment was much lower than the number who were at real risk of motor differences, yet few of these children received physiotherapy, especially in China. Motor impairment is a highly underutilized target for ASD assessment and intervention. Motor skills should be more routinely incorporated into comprehensive ASD screening, evaluation, and treatment planning. It should also be monitored beyond the early developmental period, when the focus of parents and providers often shifts away from motor milestones.

## Data availability statement

The raw data supporting the conclusions of this article will be made available by the authors, without undue reservation.

## Ethics statement

The studies involving human participants were reviewed and approved by the Institutional Review Board of the Children's Hospital of Fudan University, Shanghai. Written informed consent to participate in this study was provided by the participants' legal guardian/next of kin.

## Author contributions

XX and QX contributed to the conception of the study. BZ performed the data analyses and wrote the manuscript. BZ, HL, YZha, DL, PD, PL, and YZhu contributed to the assessments and data collections. All authors listed have made a substantial, direct, and intellectual contribution to the work and approved it for publication.

## Funding

This study was supported in part by the Key Subject Construction Project of Shanghai Municipal Health Commission (shslczdzk02903), the National Natural Science Foundation of China (NSFC, 61733011 and 82171540), and the National Key Research and Development Program of China (No. 2016YFC1306205).

## Conflict of interest

The authors declare that the research was conducted in the absence of any commercial or financial relationships that could be construed as a potential conflict of interest.

## Publisher's note

All claims expressed in this article are solely those of the authors and do not necessarily represent those of their affiliated organizations, or those of the publisher, the editors and the reviewers. Any product that may be evaluated in this article, or claim that may be made by its manufacturer, is not guaranteed or endorsed by the publisher.
